# Recruitment and retention of the rural podiatry workforce in Aotearoa New Zealand: a qualitative descriptive study of podiatrist perceptions

**DOI:** 10.1186/s13047-022-00562-3

**Published:** 2022-08-09

**Authors:** Erin Beeler, Angela Brenton-Rule, Matthew Carroll

**Affiliations:** Department of Podiatry, School of Clinical Sciences, Faculty of Health & Environmental Sciences, Auckland University of Technology, Private Bag 92006, Auckland, 1142 New Zealand

**Keywords:** Podiatry, Rural workforce, Workforce recruitment, Workforce retention

## Abstract

**Background:**

Past research into the Aotearoa New Zealand (NZ) podiatry workforce has indicated a shortage of podiatrists, particularly in rural NZ. However, there has been no research investigating the characteristics of the NZ rural podiatry workforce. This study aimed to explore the factors which contribute to recruitment and retention of primary care podiatrists in rural NZ.

**Methods:**

A qualitative descriptive approach was implemented for data collection and analysis. Semi-structured interviews were conducted with 15 podiatrists who currently, or previously, worked in a rural podiatry practice. Manifest content analysis was used to analyse participant’s responses. A deductive approach was used where data were identified and coded according to predetermined themes from the literature.

**Results:**

Four themes that influenced recruitment and retention were derived from the interviews: (1) professional factors, (2) economic factors, (3) social factors, and (4) external factors. Interviews revealed that clinical inexperience, a sole practice environment, professional and social isolation, and workload pressures combined to affect recruitment and retention. Strong community bonds, family ties, and a rural background were crucial to thrive in the rural setting.

**Conclusion:**

A sustainable rural podiatry workforce is required to reduce health disparities that exist in NZ rural communities. The study identified that most practitioners entered the rural workforce into self-employed positions, often shortly following graduation from university. They soon reported feelings of professional isolation due to limited support networks. Practitioners established in the rural workforce noted significant workload stresses. Stresses stemming from an inability to source locums, take time away from work, or recruit new staff to fill vacant positions. Research examining support mechanisms for inexperienced practitioners and targeted strategies to grow the rural workforce and reduce attrition is required.

## Background

The estimated population of Aotearoa New Zealand (NZ) (the bilingual name for NZ) as of December 2021 was 5.12 million people [1]. One in four NZ’s live in a rural area, with a greater percentage of children, older people, and Māori (the indigenous people of NZ) living rurally [1]. Approximately 19% of NZ’s population access rural healthcare [1] with 16.3% of NZ’s population is classified as living rurally [2]. 2021 NZ podiatry workforce data indicates approximately 70% of podiatrists work in major or large urban centres as classified by Statistics New Zealand functional rural–urban classification (Urban Rural Experimental Profile 2004; UREP) [2]. However, defining the NZ rural/urban population attributes for research purposes is difficult as there is no consistent agreed definition of rurality [3]. The current UREP classification of urban/rural attributes based upon population size has been criticised as the population defined as “rural” differs from that which actually receives rural health care [4].

In NZ the profession of podiatry is considered as part of the Allied Health workforce. The Allied Health workforce defined as health professionals who are not part of the medical, dental or nursing professions [5]. There are at least 43 professions that are classed as Allied Health professions in NZ. According to Allied Health Aotearoa New Zealand (2017), there are approximately 30,000 individuals who comprise the allied health workforce, making the workforce the second largest clinical professional group across NZ [6].

Workforce recruitment (identifying and filling staffing requirements) and retention (a measure of workforce length of stay) are key issues for the entire NZ rural health workforce (Allied Health professions plus medicine, nursing, and dentistry), and important to the long-term sustainability of NZ rural healthcare system [7, 8]. The ability of the NZ health sector to deliver services rurally is reduced, with health workforce shortages frequently articulated [9, 10]. Workforce shortages result in service gaps, manifesting to create health inequities [11, 12]. In NZ rural communities health inequities between Māori and non-Māori are most evident [13]. Across age groups and health conditions, rural Māori have higher mortality and morbidity, and lower life expectancy [14].

Many NZ health providers find recruitment and retention difficult. For health providers in rural settings the challenges are even greater, with fewer applicants and shorter tenures [15]. Previous work in Australia has shown that sourcing future workers from a rural background, coined the ‘rural background effect’ [16], is a strong predictor of future entry into rural practice [17]. Additionally, a desire for a rural lifestyle, being connected to the community, an enjoyable patient base, and the autonomy offered by the nature of rural work are factors shown to positively influence rural health workforce recruitment and retention [18, 19]. Conversely, poor matching of people to positions, excessive travel, issues with leave and locum access, professional isolation, increased workload, limited access to continuing professional development, limited job opportunities for partners, difficulties in building a social and professional network, and insufficient supervision are factors linked to poor retention within rural health workforces [18–22].

There is currently limited research that has investigated the sustainability of the NZ podiatry workforce. Carroll et al., who analysed the NZ podiatry workforce data between 2015 and 2019 concluded that the NZ podiatry workforce is in crisis and demonstrated there were a smaller number of podiatrists working outside of major urban cities (Auckland, Wellington, Christchurch) [23]. However, this research provided no insight into the rural podiatry workforce. There is currently limited evidence investigating what issues are faced by NZ podiatrists who work in a rural healthcare setting. Accordingly, the aim of this study was to explore the factors which contribute to recruitment and retention of rural podiatrists in NZ.

## Methods

This research used a qualitative descriptive approach with semi-structured interviews to gain insight into rural primary care podiatrists’ views around recruitment and retention of rural podiatrists in NZ [24]. Participants were registered podiatrists with current or prior experience working in a rural podiatry practice. As there is no consistent agreed definition of rurality [4], potential participants self-identified as working/having worked rurally. An email invitation to participate was sent by the Registrar of the Podiatrists Board of NZ (PBNZ) and an advertisement was posted on the NZ Podiatry Alumni Facebook page. Eligible participants were then purposively selected to achieve diversity across the following characteristics: gender, age, practice background (rural or urban) and place of podiatry education. Ethical approval was granted by the Auckland University of Technology ethics committee (AUTEC) 20/166.

Interviews were conducted via video conferencing (Zoom) or telephone by one researcher (EB). All interviews were recorded, with verbal consent obtained prior to recording, and were between 45 and 80 min in duration. Interview questions (Additional File 1) were based on previous work relating to rural recruitment and retention of healthcare workers in Australia [12]. Questions were broad and open-ended to gain a rich description of participant's thoughts, feelings, and experiences.

Data collection and analysis occurred simultaneously. As such dialogue from participant interviews influenced subsequent interviews and data analysis. Interviews continued until information power was reached [25]. Information power was determined by items such as study aim, sample specificity, use of established theory, quality of dialogue, analysis strategy and the depth of discussions during interviews [25]. Audio recordings were transcribed verbatim prior to analysis. To ensure accuracy, each transcript was listened to repeatedly, and accompanying transcripts were read and re-read to immerse the researcher in the data. Manifest content analysis was used to analyse the participant’s responses [26]. Initial analysis was undertaken by one researcher (EB) using a three-step process: preparation, organisation, and reporting [27]. A deductive approach was used where data were identified and coded according to predetermined themes from the literature: economic, professional, social, and external factors. Themes were reviewed with supporting excerpts from transcripts that accompanied each theme agreed upon by EB and ABR to represent the truthfulness of the data.

## Results

Eleven females and four males participated in this study. Fifty three percent (*n* = 8) of participants were from the North Island with the 47% (*n* = 7) aged between 30 to 55 years of age. Participant characteristics are detailed in Table 1.

**Table 1:** Participant demographics and number for reference and attribution of quotations

Participant number	Gender	Age range	Workplace setting	Geographic location
P01	Male	30–55	Rural	North Island
P02	Male	30–55	Rural	North Island
P03	Female	30–55	Rural	South Island
P04	Female	55 +	Urban^b^	North Island
P05	Female	55 +	Urban^b^	South Island
P06	Female	30–55	Rural	South Island
P07	Female	30–55	Rural	North Island
P08	Female	30–55	Rural	North Island
P09	Male	30–55	Rural	North Island
P10	Female	< 30	Rural^a^	North Island
P11	Female	< 30	Rural	North Island
P12	Female	55 +	Rural	South Island
P13	Female	55 +	Rural	South Island
P14	Female	55 +	Rural	South Island
P15	Male	55 +	Rural	South Island

^a^ Now left profession

^b^ Started in rural practice

**Fig. 1:**
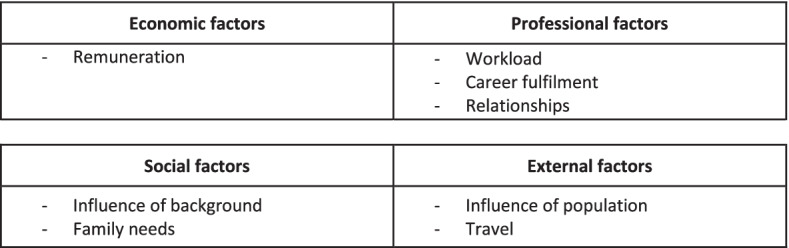
Thematic map showing the central themes and sub-themes

Four central themes were derived from the data: (1) economic, (2) professional, (3) social, and (4) external factors. The thematic schema including the central themes and sub-themes is displayed in Fig. 1.

### Theme 1 – economic factors

Economic factors were specifically related to the influence of remuneration. Most participants felt that remuneration in rural areas was either the same as what they could earn in an urban centre, or slightly better:

*“I was paid [more] because it was not an Auckland job” (Male, age 30–55, P12).*

*“[Remuneration] is a factor…[podiatrists] can earn really well here” (Male, age 30–55, P01).*

Whilst higher remuneration was an incentive, participants commented that work should not just be about money and that there were many hours of unpaid work.

*“Remuneration is great but if that was a driving force [for working rurally] then I’d be a bit nervous” (Male, age 30-55, P01).*

*“In a rural area, you put many more hours in than you actually are getting paid for” (Female, age 30–55, P03).*

### Theme 2—professional factors

Professional factors encompassed the sub themes of workload and work type, career fulfillment, and interprofessional relationships. Participants viewed the move to rural practice as an opportunity to become self-employed and an easy way to find work:

*“I was keen to work for myself, and have my own practice, and shape it” (Male, age 30–55, P01).*

*“I just thought I’d quite like to do something where I could be self-employed” (Female, age 55 + , P12).*

*“One [rural clinic] came about because no one else wanted to do it” (Male, age 30–55, P02).*

Workload was considered a significant issue by many participants with issues related to the number of hours worked and the number of podiatrists working in the rural environment common:

*“You can’t just leave that day… whatever happens that day has to be dealt with that day, because you’re in another place the next day [and] if somebody is driving for an hour and a half to see you, you can’t say oh just come back tomorrow” (Female, age 30-55, P03).*

*“There are not enough podiatrists in the area. Not even close… the more podiatrists in the area the less pressure on me!” (Female, age 55 + , P06).*

*“I never really went away… any time I had off I worked around Christmas or long weekends” (Female, age 55 + , P14).*

The issue of recruitment was twofold, firstly finding podiatrists to fill positions was difficult but also the perception that whilst they were too busy, they considered themselves not busy enough to employ additional support.

*“[We’ve been] advertising for two years and haven’t had anyone apply... I never had any trouble in Auckland hiring anyone... but it's different here, there's just not anyone to even pass the work on to” (Female, age 55*+*, P12).*

*“I don’t employ because I could only offer one or two days, and no-one would relocate for such a small amount of work. I’m too busy for just me, but not busy enough to justify employing someone full time” (Male, age 30-55, P02).*

The difficulty in recruiting new podiatrists was linked to too few graduates entering the workforce and the location of the university. Difficulties in retaining podiatrists and staff turnover also reported:

*“The shortage has to be addressed at the beginning” (Female, age 55 + , P12).*

*“If it was somewhere more central, not Auckland, Auckland is too expensive” (Female, age 55 + , P12).*

*“I had six podiatrists (including two new graduates) in the last year – one lasted 12 months, one lasted 13 and I put hours and hours into helping them” (Female, age < 30, P10).*

Participants discussed the career fulfilment gained from their interpersonal relationships with patients and the variety of work as a key to their passion for rural practice:

*“I love the people. They appreciate me, they want to get better, its relaxed and comfortable and I feel like I’m giving something back – and I didn’t get that in my urban practice” (Female, age 55 + , P14).*

*“It's one of the strengths of our community, is that we are very well connected [sic]. The referral pathways are very close... the patient's journey is... very efficient... and in terms of learning, we can get you in observing orthopedic surgery and having time with other specialists... is one of the advantages” (Male, age 30-55, P01).*

*“In the city practices people tend to find niches but here you can do a bit of everything” (Male, age 30–55, P01).*

### Theme 3—social factors

Social factors included the subthemes of influence of rural background/community affiliation and family needs. Participants commented that previous rural connections were a strong driver in the choice to work rurally:

*“That was where my family was… this became my new home” (Male, age 30–55, P01).*

*“I’m not a city person… I’m a rural person and I’m close to my family” (Female, age 55 + , P14).*

The implications related to family life and working in the rural setting were a strong pull to live and work in the rural setting. However, there were both positives and negatives to the rural environment, positives included:

*“Me and my partner can both get jobs, that pay the same anywhere, but it is way cheaper to buy a house here” (Female, age < 30, P11).*

*“I work rurally, but we live [more urban] because of [our children’s schooling]” (Female, age 30–55, P08).*

*“I love the outdoors, cost of living, cost of housing. There are so many positives” (Male, age 30–55, P01).*

A negative aspect of practicing rurally related to isolation and not being near family:

*“I’m not near family, and that’s hard” (Female, age 30–55, P07).*

*“There are lots of things that are attractive about rural areas, the people, the lifestyle etc., but it is extremely hard because you are on your own” (Female, age < 30, P11).*

*“It's quite a lonely profession, especially when you are doing it on your own… It's really nice when we get together out of work hours… because who else do you talk to?” (Female, age 55 + , P12).*

### Theme 4—external factors

External factors included the subthemes of influence of population age and travel. The composition of the community was found to be difficult for some practitioners who were new to a rural area:

*“We do have a void, a vacuum of people of 18–22 in this community and I don’t think that is unusual as they are off on OEs or at uni” (Male, age 30–55, P01).*

*“It was hard to make friends, I was very young compared to much of the population” (Female, age < 30, P10).*

Difficulties associated with work related travel was identified by most participants:

*“The main thing was the travel... and there were some safety aspects about the rural travel... especially in the wintertime because the weather can change so quickly... I had to be prepared to have my family in order and have things in my car (incase the road closed)... the roads are quite risky... I spent sometimes an hour to get to one house just to turn around and come back 20 minutes later” (Female, age 30-55, P08).*

## Discussion

This qualitative study has achieved its aims of exploring the factors that contribute to recruitment and retention of rural primary healthcare podiatrists in NZ. The findings parallel Allen et al. who found professional factors were integral in the recruitment and retainment of rural based Australian medical specialists [12]. In addition, the study found a complex interaction between economic, professional, social, and external factors which combine to affect recruitment and retention within the NZ rural podiatry workforce.

**Fig. 2:**
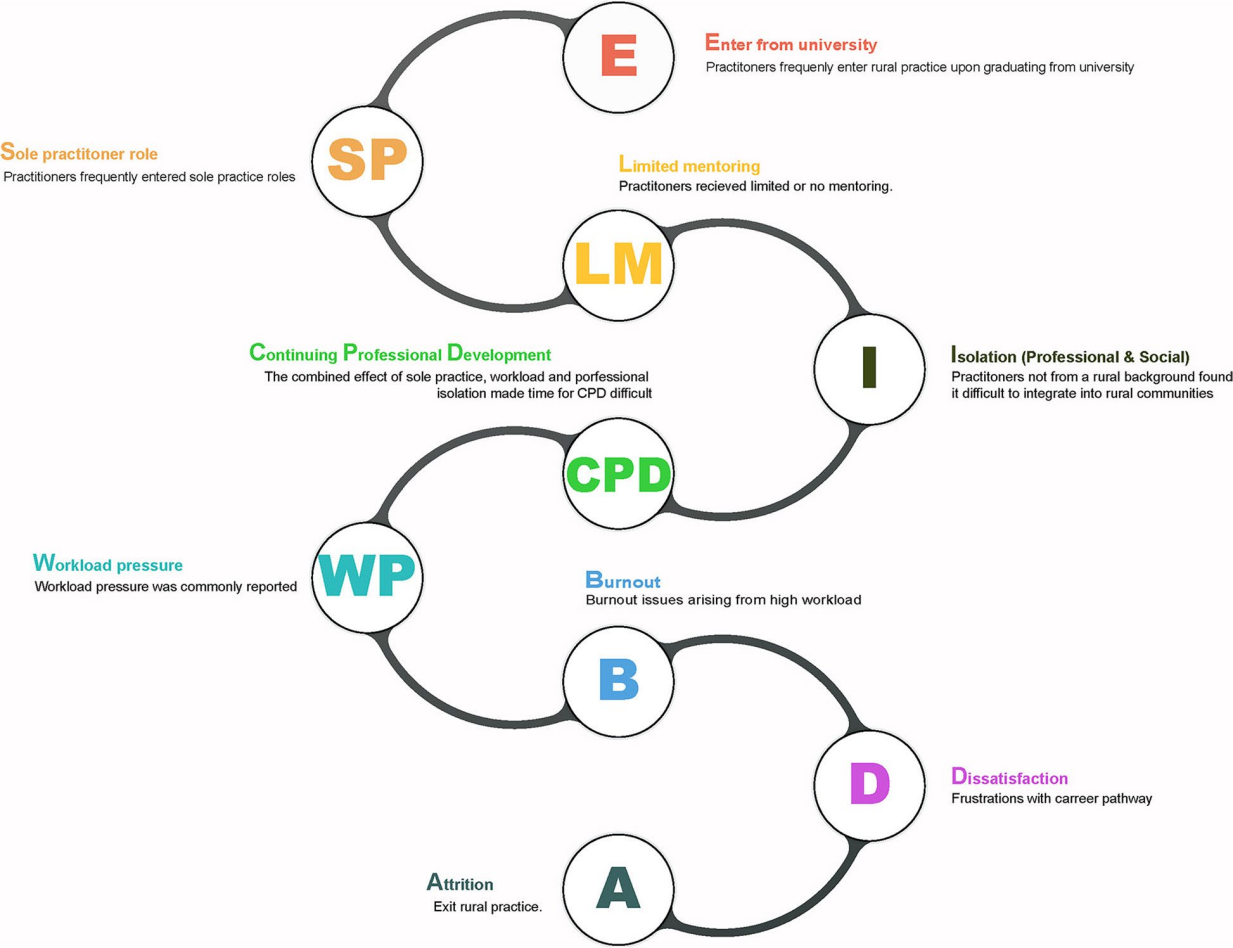
Model of attrition for NZ rural podiatry practitioners

The current study has revealed that the ability to retain new podiatry practitioners may stem from a cycle of events set in place when they first enter the rural workforce. Figure 2 summarises these events as a proposed model of rural workforce attrition. For new graduates entering the workforce the establishment of mentoring and supportive peer networks is imperative to aid both professional support and continued development [28]. However, in the current study, a feeling of professional isolation born from limited support networks was common amongst rural practitioners. Being away from immediate family, difficulty fitting in with the community, having no established network of friends, no people of similar age to socialize with, and difficulties making friends compounding the feeling of isolation. The potential effects on both professional and personal development of an inexperienced practitioner working as a sole practitioner in relative isolation could lead to negative health consequences and ultimately attrition. Professional isolation has been reported in Australian based healthcare workers as a factor that negatively influences rural workforce retention [20, 22].

For those established in the rural workforce workload stresses continued to dominate. Most notably there were inherent difficulties in sourcing locums leading to an inability to take time off. This was best surmised by the quote “there’s no time to be sick”. Interestingly, while the practitioners reported high workloads, they also reported often not trying to recruit, as they did not feel they had enough work to justify employing a second podiatrist on a full-time basis. There was also a sense that recruitment was futile as many had experienced issues with retention, often imparting a great deal of personal resources into new staff, only to have them stay for a relatively short period of time. This sentiment is echoed by the findings of Struber and Chisolm et al. who reported that the average length of stay in a rural Australian health practice was 13 -18 months [8, 20].

Consistent with previous research some positives related to rural work were identified with many thriving in the rural workforce [12, 18, 22, 29, 30]. The key enablers for this positive experience being a sense of belonging to a community, variety of clinical work, and working with an enjoyable patient base. Whilst working with other podiatrists was often not possible, professional isolation was negated by the close-knit nature of a rural healthcare team. In alignment with the above factors social/family ties were identified as a significant driver in attracting practitioners to rural positions. The availability of work for partners and the suitability of local schooling was also important in drawing practitioners to consider rural relocation.

Although the above sections largely acknowledge the difficulties encountered by practitioners surrounding the rural workforce there remains a large ‘elephant in the room’ for the NZ podiatry workforce. That is the need to create a larger rural podiatry workforce to address health inequalities in rural communities to bridge potential unmet service needs. The ability to address the shortfall of podiatrists is limited by there being only one training facility, located in Auckland. In the medium to long term, the creation of a sustainable rural podiatry workforce can only be addressed by the production of more NZ trained graduates, ideally drawn from a rural background. However, strategies to grow the workforce cannot solely focus on a simple increase in numbers. To address rural health inequities podiatrists entering the rural workforce must understand the contexts underpinning Māori health inequities and wellbeing within rural communities. With these factors identified it may be time for the podiatry undergraduate training facility to adopt a rural admissions scheme as has been implemented by two NZ medical schools [31, 32].

A key limitation is that we do not know how representative of the NZ rural workforce the participant responses were. This limitation largely stems from a lack of how ‘rurality’ should be defined. To address this limitation, rural workforce monitoring is required to provide data to accurately describe the NZ rural podiatry workforce. The study results must also be considered in light of the diversity of the population, the majority of the participants were aged 30 years or older, with the perspectives of practitioners aged less than 30 years old underrepresented. Future research is required to determine whether there is indeed an unmet service need within NZ rural podiatry. Research must gain insight into practitioners new to the rural environment and investigate strategies that facilitate peer support. The development of a programme that facilitates peer support/mentoring networks amongst rural podiatrists should be a priority for the NZ podiatry profession.

## Conclusion

A sustainable rural podiatry workforce is required to reduce health disparities that exist in NZ rural communities. The study identified that practitioners entered the rural workforce into self-employed positions, often shortly following graduation from university. They soon reported feelings of professional isolation due to limited support networks. Practitioners established in the rural workforce noted significant workload stresses. Stresses stemming from an inability to source locums, take time away from work, or recruit new staff to fill vacant positions. Research examining support mechanisms for inexperienced practitioners and targeted strategies to grow the rural workforce and reduce attrition is required.

## Data Availability

The datasets used and/or analysed during the current study are available from the corresponding author upon reasonable request.

## Abbreviations

NZ: Aotearoa New Zealand
PBNZ: Podiatrists Board of NZ
